# Consumption of carbonated soft drinks and association with health behaviours and mental health among adolescents in Malaysia: findings from 2022 Adolescent Health Survey (AHS)

**DOI:** 10.1186/s40795-025-01044-y

**Published:** 2025-03-27

**Authors:** Nor Azian Mohd Zaki, Lai Wai Kent, Syafinaz Sallehuddin, Norhafizah Sahril, Ruhaya Salleh

**Affiliations:** 1 Institute for Public Health, National Health Institute (NIH), Seksyen U13 Setia Alam, Jalan Setia Murni U13/52, Seksyen U13 Setia Alam, Shah Alam, Selangor 40170 Malaysia; 2 Raub Health Clinic, Jalan Cheroh Lama, Raub, Pahang 27600 Malaysia

**Keywords:** Carbonated soft drink, Adolescent Health Survey (AHS), Health behaviour, Mental health

## Abstract

**Background:**

The consumption of carbonated soft drinks has risen substantially and has been associated with weight gain, chronic disease, and oral health issues. This study seeks to examine the relationship between carbonated soft drink consumption, health behaviours and mental health among Malaysian adolescents.

**Methods:**

The 2022 Adolescent Health Survey (AHS) 2022 was a nationwide study involving Malaysian students aged 13 to 17 years old. It used a multistage stratified cluster sampling method to ensure a representative sample. Data collection was performed via a validated self-administered questionnaire. Descriptive and multivariate logistic regression was used to determine the prevalence and factors associated with the consumption of carbonated soft drinks.

**Results:**

The prevalence of carbonated soft drink consumption at least once daily in the past 30 days was 32.4% (95% CI: 30.93, 33.87). The results revealed that the consumption of carbonated soft drinks was highest among males and young adolescents. Multivariate logistic regression analysis indicated that health-related behaviours associated with high soft drink consumption included consuming fast food at least three days per week, drinking alcohol, drinking fewer than six glasses of plain water, and being physically inactive. In addition, a greater intake of carbonated soft drinks was positively associated with depression and suicide attempts.

**Conclusion:**

This study provides compelling evidence that the consumption of carbonated soft drinks was associated with health-related behaviours and poor mental health among Malaysian adolescents.

## Introduction

In recent decades, soft drink consumption has risen significantly, with children and adolescents seeing the largest increase. The Global School-based Student Health Survey (GSHS), conducted from 2009 to 2013 found that 54.3% of adolescents in 53 lower-middle-income countries drank carbonated beverages at least once a day [1]. A study conducted in 18 nations across the Americas, Asia, and Africa reported that the overall prevalence of carbonated soft drink consumption among 74,055 students aged 12 to 15 years old was 42.1% [2]. Additionally, a study in six Southeast Asian countries revealed that approximately 20% of adolescents consume sugar-sweetened carbonated soft drinks once daily, while around 18% consume more than twice a day [3]. The Malaysian Global School-based Health Survey data in 2012 indicated that 29.3% of 13 to 17 year old adolescents consumed carbonated drinks at least once daily, which increased to 36.9% in 2017 [4, 5].

The consumption of sugar-sweetened beverages (SSBs), such as carbonated soft drinks, cordials, and sweetened fruit drinks has detrimental effects on overall health. The SSBs are non-alcoholic drinks that have been extensively studied for their ability to increase the risk of conditions such as diabetes mellitus, metabolic syndrome, osteoporosis, and cancer, as well as their negative impacts on oral health [6–8]. Increased soft drink consumption which is linked to higher calorie intake, contribute to obesity and excess weight in children as well as adults [9–11]. Moreover, the excessive intake of beverages that are high in sugar and acidity can adversely affect dental health, contributing to the development of cavities and tooth erosion [12].

Considering the negative impacts of excessive carbonated beverage consumption on health, it is crucial to understand the factors influencing adolescents' consumption of soft drinks to develop effective interventions. Many studies have linked soft drink consumption with dietary habits and mental well-being. In a study of Australian high school students, drinking soft drinks was strongly associated with eating less fruit, having fast food once a week, snacking more than 14 times per week, watching over 2 h of television daily and getting less than 8 h of sleep on school nights [13].

A cross-sectional study found that soft drink consumption was linked to mental health issues and risky health behaviours, such as skipping school and using tobacco and alcohol [3]. In addition, a higher intake of carbonated soft drinks was linked to a history of loneliness, anxiety, suicidal ideation, suicide planning, and suicide attempts [14, 15]. Another study revealed that adolescents who consumed carbonated beverages more than seven times weekly, or exceeded 25 g of sugar per day from such beverages, exhibited notably elevated levels of depression and anxiety [16].

In Malaysia, there exists a scarcity of research examining the relationship between the intake of carbonated soft drinks, health-related behaviors, and the mental well-being of adolescents. As the prevalence of non-communicable diseases and mental health concerns among young individuals continues to rise, it is essential to understand these relationships in order to formulate effective targeted interventions. This study aims to examine the associations between soft drink consumption and factors such as socio-demographic characteristics, health behaviors, and mental health using data from the 2022 Adolescent Health Survey (AHS). The findings will provide valuable insights into the behavioral and psychological factors influencing soft drink consumption, helping policymakers and healthcare professionals allocate resources and design effective interventions to promote healthier habits among adolescents.

## Methods

### Study design and sampling

The Adolescent Health Survey was a cross-sectional study carried out among school-going adolescents aged 13 to 17 in selected secondary schools, using a twostage stratified cluster sampling method. The sampling frame comprises 2798 national secondary schools that were officially registered in 2021, encompassing both governmental and private institutions which under the jurisdiction of the Ministry of Education. The first step involved selecting secondary schools from all eligible schools (*N* = 2798) across Malaysia. A total of 240 schools were randomly chosen using probability proportional to enrollment across all forms. Within each state, 16 schoolss were selected through a random process, with the exception of the two small federal territories, which each had 8 schools chosen. In the second step, classes from each form were selected using systematic probability sampling. The number of classes in each school was determined based on the sampled schools and student enrollment. All the students from the selected classes were eligible and asked to participate in the survey. A detailed explanation of this study methodology has been provided elsewhere [17].

### Study instrument

All the questionnaires used were derived from the Global School-based Student Health Survey (GSHS), developed by the World Health Organization (WHO [18]. Data collection was done via validated self-administered bilingual questionnaires (Bahasa Melayu and English) to ensure clarity and accessibility for all participants. The answers were provided via separate answer sheets to maintain anonymity and confidentiality. The questionnaires cover ten main topics, including health risk behaviours and mental health.

### Data collection

Data collection was conducted for two months, between June and July 2022. The information sheets were given to all the selected students and their parents or guardians. Written informed consent was obtained from the student's parents or guardians before the survey began. Additionally, the participating students signed a consent form before completing the questionnaire. Only adolescents who had obtained consent from both themselves and their parents were included in the study. Ethical approval was granted by the Medical Research Ethics Committee (MREC), Ministry of Health, Malaysia (NMRR-21–157-58,261). Approval for the study was also secured from the Ministry of Education at the national, state, and school tiers.

### Variable definition

Sociodemographic variables such as gender (male, female), age (grouped into 2 categories: 13 to 14 years old and 15 to 17 years old), and ethnicity of the students were collected during the survey. Ethnicity was classified according to the primary ethnic groups in Malaysia including Malays, Chinese, Indians, Bumiputra Sarawak, Bumiputra Sabah and others.

The dependent variable, carbonated soft drinks consumption, was evaluated using the following question; “During the past 30 days, how many times per day did you usually drink carbonated soft drinks, such as Coca-Cola, Sprite, and Pepsi? (Do not include diet soft drinks). Carbonated soft drink consumption (times per day) was determined by converting responses indicating never or less than once per day to “0” and once per day or more to “1”.

The independent variables explored in this study were socio-demographic characteristics, body weight status, health behaviour and mental health. The respondents’ body weight and height were measured twice by trained research team members using electronic weighing scales TANITA-HD 319 (Tanita Corporation, Tokyo, Japan) and SECA 213 stadiometer (SECA GmbH & Co. KG, Hamburg, Germany). The WHO Anthro Plus software was used to calculate body mass index (BMI) for age based on the average values of body weight and body height. Weight status was classified into thin, normal, overweight, or obese according to the WHO 2007 Growth Reference Data for individuals aged 5 to 19 years (World Health Organization, 2007) [19].

This study looked at health behaviours that may be linked to soft drink consumption, including dairy products, water consumption, fast food habits, alcohol use and physical activity levels. Physical activity was assessed using with the question; “During the past 7 days, on how many days were you physically active for a total of at least 60 min per day?” and the options provided ranged from zero to seven days. Adolescents who engaged in at least 60 min of physical activity per day, for at least five days per week were considered physically active.

Other factors that were investigated in this study were mental health factors, which included depression, loneliness, suicidal ideation, suicide plans and suicide attempts. A validated Patient Health Questionnaire (PHQ-9) was used to measure depression, with a score of 10 or higher indicating the presence of depression [20]. Loneliness was determined with an answer indicating feeling lonely "most of the time or "always". Suicide ideation and suicide plans were evaluated with the following questions; “During the past 12 months, did you ever seriously consider attempting suicide? and “During the past 12 months, did you make a plan about how you would attempt suicide?" with the response "yes" or "no" respectively. The assessment for suicide attempts was determined using the following question; "How many times did you attempt suicide in the past 12 months?" and defined it as at least one attempt during this period.

### Statistical analysis

The data were analysed utilizing SPSS version 26. (SPSS IBM, New York, USA). Prior to analysis, the data were adjusted according to the study design and nonresponse rate. Descriptive statistics were employed to outline the population characteristics and determine the prevalence of carbonated soft drink consumption. Univariate and multiple logistic regression analyses were conducted to examine the relationship with the dependent variables (carbonated soft drink consumption) and the independent variables (sociodemographic characteristics, body weight status, health behaviour and mental health). All tested variables at *P*-value < 0.25 were included in the final model. A diagnostic test for goodness of fit was performed on the logistic regression model. The analysis results were described using 95% confidence intervals, and a *p*-value < 0.05 was deemed to indicate statistical significance.

## Results

A total of 33,523 students completed the questionnaire, yielding a response rate of 89.4%. Approximately 32.4% of Malaysian adolescents reported consuming carbonated soft drinks at least once per day in the past 30 days. Male and younger adolescents consumed carbonated soft drinks more frequently than female and older adolescents (34.3% versus 30.5%) and (36.0% versus 29.7%), respectively. Bumiputra Sarawak adolescents had the highest prevalence rate of carbonated soft drink consumption, while Chinese adolescents had the lowest. The intake of carbonated soft drinks was highest among those who drank plain water less than 6 glasses per day, had fast food for at least 3 days per week, and drank alcohol, as revealed in Table 1.

**Table 1:** Prevalence of carbonated soft drink intake at least once a day in the past 30 days among adolescents

**Variable**		**Unweighted count**	**Prevalence (%)**	**95% Confidence Interval**	
				**Lower Limit**	**Upper Limit**
Malaysia		10,614	32.4	30.93	33.87
Gender	Male	5235	34.3	32.6	36.0
	Female	5379	30.5	29.0	32.0
Age	13–14 years	4924	36.0	34.3	37.8
	15–17 years	5690	29.7	28.1	31.5
Ethnicity	Malay	7139	32.1	30.8	33.4
	Chinese	1101	21.4	18.8	24.3
	Indian	535	35.7	32.2	39.3
	Bumiputera Sabah	753	42.1	37.4	46.9
	Bumiputera Sarawak	680	55.8	50.8	60.8
	Others	352	43.2	38.5	48.0
BMI for age	Thinness/ Normal	7434	32.9	31.3	34.5
	Overweight/Obese	3157	31.3	29.5	33.1
Plain water intake	Less than 6 glasses daily	5572	35.1	33.5	36.8
	6 glasses or more per day	5038	29.7	28.2	31.3
Dairy Products	Less than 2 times per day	6967	28.2	26.8	29.6
	At least 2 times per day	3639	46.3	44.3	48.3
Fast Food Intake	Less than 3 days per week	8788	30.2	28.8	31.7
	At least 3 days per week	1823	51.0	48.5	53.4
Alcohol drinker	Yes	820	41.1	36.9	45.5
	No	9697	31.6	30.2	33.0
Physically active	Yes	2203	31.3	29.5	33.1
	No	8403	32.7	31.1	34.3
Depression	Yes	3451	39.0	37.0	41.1
	No	7121	29.9	28.5	31.4
Loneliness	Yes	2087	38.6	36.4	40.9
	No	8525	31.2	29.8	32.6
Suicidal ideation	Yes	1689	39.3	36.6	42.0
	No	8921	31.3	29.9	32.8
Suicidal plan	Yes	1322	40.6	38.0	43.2
	No	9285	31.5	30.0	32.9
Suicidal attempts	Yes	1378	44.3	41.7	46.8
	No	9229	31.1	29.7	32.5

Table 2 outlines the factors independently associated with drinking carbonated soft drinks at least once daily. A simple logistic regression analysis indicated that all the factors examined were significantly associated with carbonated soft drinks consumption, except for weight status and physical activity. Multivariate logistic analysis revealed that the variances in prevalence rates were consistent those in with males (aOR: 1.41; 95% CI: 1.32–1.51), younger adolescents aged 13 to14 years old (aOR: 1.35; 95% CI: 1.24–1.46) and adolescent who were thin and had normal BMI (aOR: 1.11; 95% CI: 1.03–1.20) had higher odds of consuming carbonated drinks. In terms of health-related behaviors, it has been observed that adolescents who reported their plain water consumed fewer than six glasses daily (aOR: 1.32; 95% CI: 1.24–1.40), consumed fast food at least three days per week (aOR: 2.05; 95% CI: 1.86–2.26), drank alcohol (aOR: 1.49; 95% CI: 1.31–1.70) and were physically inactive (aOR: 1.17; 95% CI: 1.08–1.28) were more likely to be high soft drink consumers compared to their counterpart. The odds of high soft drink intake were also greater among adolescents who reported having depression (aOR: 1.30; 95% CI: 1.20–1.41) and had attempted suicide (aOR: 1.40; 95% CI: 1.26–1.57).

**Table 2:** Factors associated with carbonated soft drink intake among adolescents

Variable	Characteristic	Crude OR (95% CI)	*p*-value	Adjusted OR (95% CI)	*p*-value
Gender	Male	1.19 (1.12, 1.27)	< 0.001	1.41 (1.32,1.51)	< 0.001
	Female	1.00		1.00	
Age	13–14 years	1.33 (1.22, 1.45)	< 0.001	1.35 (1.24,1.46)	< 0.001
	15–17 years	1.00		1.00	
Ethnicity	Malay	1.73 (1.47, 2.05)	< 0.001	1.69 (1.42, 2.01)	< 0.001
	Chinese	1.00		1.00	
	Indian	2.04 (1.68, 2.47)	< 0.001	1.96 (1.60, 2.40)	< 0.001
	Bumiputera Sabah	2.67 (2.09, 3.41)	< 0.001	2.64 (2.10, 3.37)	< 0.001
	Bumiputera Sarawak	4.64 (3.59, 5.99)	< 0.001	4.27 (3.30, 5.52)	< 0.001
	Others	2.78 (2.19, 3.53)	< 0.001	2.65 (2.09, 3.37)	< 0.001
BMI for age	Thinness/Normal	1.07 (0.99, 1.16)	0.058	1.11 (1.03–1.20)	0.009
	Overweight/Obese	1.00		1.00	
Plain water intake	Less than 6 glasses daily	1.28 (1.20, 1.36)	< 0.001	1.32 (1.24, 1.40)	< 0.001
	6 glasses or more per day	1.00		1.00	
Dairy Products	Less than 2 times per day	1.00		1.00	
	At least 2 times per day	2.19 (2.05, 2.36)	< 0.001	2.10 (1.94–2.25)	< 0.001
Fast Food Intake	Less than 3 days per week	1.00	< 0.001	1.00	
	At least 3 days per week	2.40 (2.18, 2.65)		2.05 (1.86, 2.26)	< 0.001
Alcohol drinker	Yes	1.51 (1.29, 1.77)	< 0.001	1.49 (1.31, 1.70)	< 0.001
	No	1.00		1.00	
Physically active	Yes	1.00		1.00	
	No	1.07 (0.99, 1.15)	0.108	1.17 (1.08, 1.28)	0.002
Depression	Yes	1.49 (1.40, 1.59)	< 0.001	1.30 (1.20, 1.41)	< 0.001
	No	1.00		1.00	
Loneliness	Yes	1.39 (1.59, 1.49)	< 0.001	1.04 (0.95,1.13)	0.404
	No	1.00		1.00	
Suicidal ideation	Yes	1.42 (1.30, 1.55)	< 0.001	1.04 (0.92, 1.19)	0.513
	No	1.00		1.00	
Suicidal plan	Yes	1.48 (1.37, 1.62)	< 0.001	1.03 (0.91, 1.56)	0.646
	No	1.00		1.00	
Suicidal attempts	Yes	1.76 (1.63, 1.89)	< 0.001	1.40 (1.26, 1.57)	< 0.001
	No	1.00		1.00	

*BMI* Body mass index, *OR* Odd Ratio

## Discussion

The current study found that over a third of the adolescents drank carbonated soft drinks daily in the past 30 days. Males and younger adolescents were more likely to consume carbonated soft drinks. Frequent consumption of carbonated soft drinks was associated with health behaviours, including less plain water intake, alcohol consumption and physical inactivity. The present study also revealed a positive association between mental health issues and carbonated soft drink intake.

The current prevalence of carbonated soft drinks consumption among Malaysian adolescents is slightly lower than that in a previous study [21], but still higher than studies from other Asian countries like Indonesia and Thailand [22]. Other local studies revealed that the consumption of carbonated soft drinks ranged from 38.0% to 51.6% among young adults [23, 24]. A meta-analysis conducted between 2008 and 2015 found that about 40% of adolescents aged 12 and 15 drank carbonated soft drinks at least once a day, with the highest consumption in the United States, followed by Africa [25].

Our findings showed that male and younger adolescents are more likely to consume soft drinks than female and older adolescents, consistent with findings from previous studies in Malaysia and other countries [21, 26]. Teng et al*.* found that female adolescents demonstrated higher awareness and a more conscientious approach to reducing sugar-sweetened beverage consumption, particularly in relation to its effects on body weight [27]. Younger adolescents are at an age where they gain more control over their food choices and are significantly influenced by marketing strategies, particularly regarding price, packaging, and branding [28]. The availability and variety of soft drinks at most convenience stores may influence the consumption of carbonated soft drinks, especially among younger adolescents. However, these findings differ from those in Germany, where sugary soft drinks consumption increases with age and is highest among older adolescents [29].

This study matches other research showing that unhealthy habits like not drinking enough water, eating too much fast food, drinking alcohol and being inactive are linked to drinking carbonated soft drink [13, 30]. The fact that soft drinks and fast food are frequently purchased or served together may explain why soft drinks and fast-food consumption were correlated. A study by Ashdown-Franks et al*.* found that the consumption of fast food and carbonated soft drinks rose with higher levels of sedentary activity per day [31]. In addition, studies have revealed a strong relationship between unhealthy dietary habits and screen-based sedentary behaviours among adolescents, such as watching TV, using a computer and playing e-games [13, 32].

The present study demonstrated that the consumption of carbonated beverages was also linked with mental health issues, including depression and increased risk of suicide attempts. Consistent with earlier studies, a positive link was observed between carbonated soft drink consumption and depression as well as mental health issues [33, 34]. Simultaneously, another study showed that soft drink consumption was associated with a greater risk of depressive symptoms among younger adolescents and boys [35]. The meta-analysis indicated that high consumption of sugary drinks in adolescents was linked to a higher risk of depression and may involve various biological factors [36]. Additionally, a multinational study found a positive link between carbonated soft drinks consumption and suicide attempts, with the strongest association in upper-middle-income countries [14]. In contrast, the results contradict a long-term study that found no connection between soft drink consumption and depression or mental health [37]. Thus, further detailed longitudinal and experimental studies are necessary to understand better the connection between carbonated soft drink consumption and mental health in adolescents.

Several initiatives have been implemented in Malaysia by the Ministry of Health (MOH) and Ministry of Education (MOE) to enhance the availability of nutritious food items at the school canteen and promote healthy eating habits among school students in Malaysia. The Healthy School Canteen Guidelines was implemented to prohibit the sale of sugary beverages, including carbonated soft drinks, within the school premises [38]. Additionally, the Malaysia National Plan of Action for Nutrition (NPANM) includes strategies to educate students on the risks of excessive sugar consumption and advocate for water consumption as a healthier alternative [39]. Despite these initiatives, challenges persist, particularly in enforcing regulations outside school hours. Adolescents can easily access carbonated soft drinks from convenience stores and vending machines, limiting the effectiveness of school-based policies. Formulating more holistic approaches that extend beyond educational institutions and intensifying public awareness initiatives regarding the detrimental health consequences of sugary beverages will be crucial in confronting this challenge.

## Strength and limitations

The major strength of this study is its large, nationally representative sample of Malaysian adolescents. This survey also utilized a standard WHO questionnaire, offering the benefit of facilitating international comparisons while ensuring respondent anonymity to encourage honest disclosure. However, the current findings should be considered in the context of several limitations. Firstly, the cross-sectional design of this study limits our ability to draw causal conclusion from the data. Secondly, the findings rely on self-reported data, which may be affected by recall bias and social desirability. Third, some concepts in this study were evaluated using single items, such as soft drink consumption, rather than a more thorough dietary assessment tool. Future research is suggested to include more comprehensive assessments.

## Conclusion

The findings of this study will support in developing intervention programs and identifying adolescents who could benefit from initiatives to reduce sugar-sweetened beverages consumption. Strategies may include implementing policies to limit advertising targeted at adolescents, improving access to healthier beverage options in schools and communities, raising awareness about the health risks associated with soft drink and promoting healthy mental well-being. Stronger collaboration is needed among policymakers, educational institutions, parents/caregiver and the food industry to ensure sustainable success in reducing the consumption of sugar-sweetened beverages among adolescents.

## Data Availability

No datasets were generated or analysed during the current study.
